# Draft genome sequences of *Bradyrhizobium shewense* sp. nov. ERR11^T^ and *Bradyrhizobium yuanmingense* CCBAU 10071^T^

**DOI:** 10.1186/s40793-017-0283-x

**Published:** 2017-12-05

**Authors:** Aregu Amsalu Aserse, Tanja Woyke, Nikos C. Kyrpides, William B. Whitman, Kristina Lindström

**Affiliations:** 1 Department of Environmental Sciences, University of Helsinki, Helsinki, Finland; 2 Joint Genome Institute, Walnut Creek, USA; 3 Department of Microbiology, Biological Sciences, University of Georgia, Athens, USA

**Keywords:** *Bradyrhizobium shewense* sp. nov. ERR11^T^, *Erythrina brucei*, *Bradyrhizobium yuanmingense* CCBAU 10071^T^, Symbiotic, Ethiopia, Genome average nucleotide identity, Digital DNA-DNA hybridization

## Abstract

The type strain of the prospective 10.1601/nm.30737 sp. nov. ERR11^T^, was isolated from a nodule of the leguminous tree *Erythrina brucei* native to Ethiopia. The type strain 10.1601/nm.1463 10.1601/strainfinder?urlappend=%3Fid%3DCCBAU+10071 ^T^, was isolated from the nodules of *Lespedeza cuneata* in Beijing, China. The genomes of ERR11^T^ and 10.1601/strainfinder?urlappend=%3Fid%3DCCBAU+10071 ^T^ were sequenced by DOE–JGI and deposited at the DOE–JGI genome portal as well as at the European Nucleotide Archive. The genome of ERR11^T^ is 9,163,226 bp in length and has 102 scaffolds, containing 8548 protein–coding and 86 RNA genes. The 10.1601/strainfinder?urlappend=%3Fid%3DCCBAU+10071 ^T^ genome is arranged in 108 scaffolds and consists of 8,201,522 bp long and 7776 protein–coding and 85 RNA genes. Both genomes contain symbiotic genes, which are homologous to the genes found in the complete genome sequence of 10.1601/nm.24498 10.1601/strainfinder?urlappend=%3Fid%3DUSDA+110 ^T^. The genes encoding for nodulation and nitrogen fixation in ERR11^T^ showed high sequence similarity with homologous genes found in the draft genome of peanut–nodulating 10.1601/nm.27386 10.1601/strainfinder?urlappend=%3Fid%3DLMG+26795 ^T^. The nodulation genes *nolYA-nodD2D1YABCSUIJ-nolO-nodZ* of ERR11^T^ and 10.1601/strainfinder?urlappend=%3Fid%3DCCBAU+10071 ^T^ are organized in a similar way to the homologous genes identified in the genomes of 10.1601/strainfinder?urlappend=%3Fid%3DUSDA+110 ^T^ *,* 10.1601/nm.25806 10.1601/strainfinder?urlappend=%3Fid%3DUSDA+4 and 10.1601/nm.1462 10.1601/strainfinder?urlappend=%3Fid%3DCCBAU+05525. The genomes harbor *hupSLCFHK* and *hypBFDE* genes that code the expression of hydrogenase, an enzyme that helps rhizobia to uptake hydrogen released by the N_2_-fixation process and genes encoding denitrification functions *napEDABC* and *norCBQD* for nitrate and nitric oxide reduction, respectively. The genome of ERR11^T^ also contains *nosRZDFYLX* genes encoding nitrous oxide reductase. Based on multilocus sequence analysis of housekeeping genes, the novel species, which contains eight strains formed a unique group close to the 10.1601/nm.25806 branch. Genome Average Nucleotide Identity (ANI) calculated between the genome sequences of ERR11^T^ and closely related sequences revealed that strains belonging to 10.1601/nm.25806 branch (10.1601/strainfinder?urlappend=%3Fid%3DUSDA+4 and 10.1601/strainfinder?urlappend=%3Fid%3DCCBAU+15615), were the closest strains to the strain ERR11^T^ with 95.2% ANI. Type strain ERR11^T^ showed the highest DDH predicted value with 10.1601/strainfinder?urlappend=%3Fid%3DCCBAU+15615 (58.5%), followed by 10.1601/strainfinder?urlappend=%3Fid%3DUSDA+4 (53.1%). Nevertheless, the ANI and DDH values obtained between ERR11^T^ and 10.1601/strainfinder?urlappend=%3Fid%3DCCBAU+15615 or 10.1601/strainfinder?urlappend=%3Fid%3DUSDA+4 were below the cutoff values (ANI ≥ 96.5%; DDH ≥ 70%) for strains belonging to the same species, suggesting that ERR11^T^ is a new species. Therefore, based on the phylogenetic analysis, ANI and DDH values, we formally propose the creation of 10.1601/nm.30737 sp. nov. with strain ERR11^T^ (10.1601/strainfinder?urlappend=%3Fid%3DHAMBI+3532 ^T^=10.1601/strainfinder?urlappend=%3Fid%3DLMG+30162 ^T^) as the type strain.

**Electronic supplementary material:**

The online version of this article (10.1186/s40793-017-0283-x) contains supplementary material, which is available to authorized users.

## Introduction

Biological nitrogen fixation is a vital process in ecosystem functioning, offering a nitrogen for plant growth. Legume plants form a nitrogen–fixing symbiotic association with soil bacteria known as rhizobia. The symbiotic association results in the formation of nodules, shelter and powerhouse of nitrogen fixation for the rhizobia, on the roots or stems of host legumes [1]. The rhizobia belong to 10.1601/nm.809 and 10.1601/nm.1616 [2]. alphaproteobacterial 10.1601/nm.1459 was first described as slow–growing rhizobia by Jordan [3]. Since then, 33 distinct rhizobial species belonging to the genus 10.1601/nm.1459 were formally described [4]. In addition, unique 10.1601/nm.1459 groups isolated from diverse legume species might represent new species [5–11].

In rhizobial taxonomic studies, polyphasic approaches such as phenotypic features, analysis of the 16S rRNA genetic marker, and DDH were for years used as standard criteria for the description of new bacterial species. Nevertheless, the 16S rRNA gene sequence difference between closely related species, particularly in the genus 10.1601/nm.1459 is low for differentiation of closely related species [5, 12, 13]. Bacterial strains in the same species could be delineated at ≥70% DDH relatedness [14, 15], but yet this method is vulnerable to variable laboratory results that lead to an inconsistent classification of the same species [16]. To resolve the issues related to the traditional wet–lab DDH technique, a digital DDH method was proposed for calculation of the DDH from genome sequences for bacterial classification study [17–19].

Multilocus sequence analysis (MLSA) of housekeeping protein–coding genes has become a common practice in bacterial taxonomic studies. The method offers high resolution and hence, has been used in rhizobial taxonomic studies for species identification and differentiating strains at the species level [5, 13, 20, 21]. Recently, the genome–wide average nucleotide Identity (ANI) method has successfully been used for classification of various bacterial species [22–24]. According to Richter and Rosselló*-*Móra [25] and Kim et al. [23], the ANI cutoff value that corresponds to the traditional 70% DNA–DNA relatedness cutoff value for species delineation was in the range 95–96%, depending on the nature of bacterial genome sequences. A more advanced ANI calculation was carried–out by Varghese et al. [24] by including a large number of genome sequences. Based on this study, a 96.5% ANI value is the minimum threshold that corresponds to 70% DNA–DNA relatedness cutoff value for strains (genomes) belong to the same species. To set the 96.5% ANI cutoff value for species description, the alignment fraction (AF) between the genomes should be 0.6 or above (i.e. AF covering at least 60% of the gene content of a pair of genomes) [24].

In Ethiopia, an endemic multipurpose legume tree *E. brucei* [26] is used for the production of firewood and a shade for coffee plantations [27] and it also improves soil fertility [28]. *Crotalaria* spp. [29] and *Indigofera* spp. [30] are among the diverse perennial herb and shrub legumes found in Ethiopia [31]. *Crotalaria* spp. [29] are used for green manuring, as a fallow before the main crop or for intercropping with cereal plants in order to amend soil nitrogen fertility. Some *Crotalaria* spp. [29] can be used as food and feed [32–34]. *Indigofera* spp. [30] are used for fodder for livestock, particularly in dryland areas as the species are resistant to water stress [35]. A group of rhizobial strains belonging to the genus 10.1601/nm.1459 was isolated from nodules of the legume tree *E. brucei* [26] and the shrub legumes *Crotalaria* spp. [29] and *Indigofera* spp. [30] growing in Ethiopia. These bacteria formed a unique branch which was distinct from other known species of the genus 10.1601/nm.1459 in phylogenetic trees constructed based on sequence analysis of housekeeping genes [5]. To describe this group as a new 10.1601/nm.1459 species using the genome–wide ANI and digital DDH methods, a representative strain 10.1601/nm.1459 sp. ERR11 (hereafter 10.1601/nm.30737 sp. nov. ERR11^T^) was selected for genome sequencing. The sequencing was done under the DOE–JGI 2014 Genomic Encyclopedia of Type Strains, Phase III, a project designed for sequencing of soil and plant–associated and newly described type strains [36]. Therefore, the main purpose of this study was 1) to present classification and general features of 10.1601/nm.30737 sp. nov., 2) to report the genome sequence and annotation of the type strain ERR11^T^. In addition, the genome sequence and annotation of reference type strain 10.1601/nm.1463 10.1601/strainfinder?urlappend=%3Fid%3DCCBAU+10071 ^T^ [37] sequenced for this study will be reported.

## Organism information

### Classification and features

**Fig. 1:**
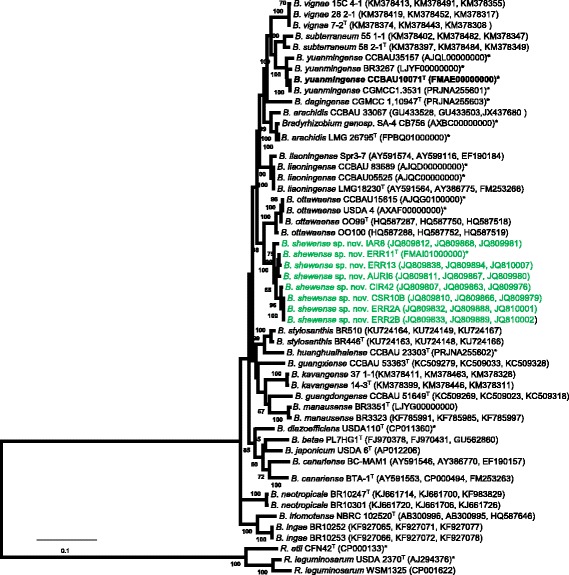
Maximum Likelihood phylogenetic tree reconstructed based on *recA-glnII-rpoB* concatenated nucleotide sequences, showing the relationships between 10.1601/nm.30737 sp. nov. (in green) and recognized species of the genus 10.1601/nm.1459 as well as the position of type strain 10.1601/nm.1463 10.1601/strainfinder?urlappend=%3Fid%3DCCBAU+1007 ^T^.The tree was constructed by using General Time Reversible model using MEGA version 7. A discrete Gamma distribution was used to model evolutionary rate differences among sites (5 categories (+G, parameter = 0.2999). The rate variation model allowed for some sites to be evolutionarily invariable ([+I], 31.7544% sites). Bootstrap values (100 replicates) are indicated at the branching points. Reference type strains are indicated with superscript ‘T’. Bar, % estimated substitutions. GenBank accession numbers of the sequences (*recA, glnII*, *rpoB* in order) are listed in parentheses next to the strains codes. The accession numbers of whole genome sequenced strains are indicated with bold*. Abbreviations: B, 10.1601/nm.1459; R, 10.1601/nm.1279; sp., species

The strain ERR11^T^ is the type strain of newly proposed 10.1601/nm.30737 sp. nov. This novel species includes strains isolated from nodules of *E. brucei* [26], *Indigofera* spp. [30] and *Crotalaria* spp. [29] growing in Ethiopia. Previously, the strains were identified as a unique group using *recA*, *glnII,* and *rpoB* single gene sequence analysis and on the phylogenetic tree constructed based concatenated *recA–glnII–rpoB* gene sequences. On the phylogenetic tree, the strains in the novel group formed their own cluster exclusive of validly published species, and consequently, this group were designated as 10.1601/nm.1459 genosp ETH1 [5]. To define the current taxonomic position of the novel rhizobial species, we reconstructed a phylogenetic tree from concatenated *recA–glnII–rpoB* sequences by including more and recently published reference sequences from the public database. In this phylogenetic tree, the bacterial grouping was consistent with our previous tree produced from concatenated *recA, rpoB* and *glnII* gene sequences [5]. The novel species formed a distinct group close to a 10.1601/nm.25806 branch that contains strains isolated from the nodules of soybean (*Glycine max*) [38] grown in Ottawa, Canada [39] (Fig. 1)*.* The average *recA–glnII–rpoB* gene sequences (1411 bp) similarity between the type strain ERR11^T^ and other strains in the novel species was in the range 99–100% (data not shown). The closest species was 10.1601/nm.25806 [39] followed by 10.1601/nm.1462 [40]*.* The similarity between strains in the novel group and strains in the closest species was 96% and they all showed 95% average gene sequence similarity with strains in 10.1601/nm.1462 (Table 5). The type strain ERR11^T^ showed 94–95% similarity of *recA–glnII–rpoB* gene sequence with the type strains of neighbor branches; 10.1601/nm.1463 10.1601/strainfinder?urlappend=%3Fid%3DCCBAU+10071 ^T^ [37], 10.1601/nm.23808 10.1601/strainfinder?urlappend=%3Fid%3DCGMCC+1.10947 ^T^ [41], 10.1601/nm.27386 10.1601/strainfinder?urlappend=%3Fid%3DLMG+26795 ^T^ [42, 43] and 10.1601/nm.27430 58 2–1^T^ [44].

**Fig. 2:**
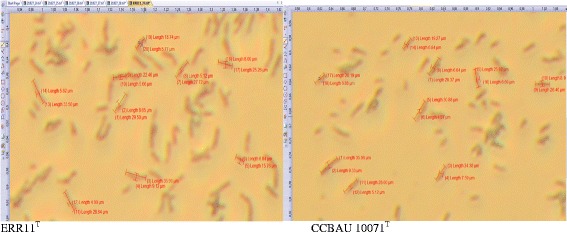
Gram stain and dimensions of 10.1601/nm.30737 sp. nov. ERR11^T^ and 10.1601/nm.1463 CBAU1007^T^

Minimum Information about the Genome Sequence is provided in Table 1 and the Additional file 1: Table S1. The type strain ERR11^T^ is a rod–shaped Gram–negative strain and has a dimension of 1.0–2.3 μm length and 0.7–1.0 μm width (Fig. 2). The species includes slow–growing bacteria, forming creamy, raised, smooth margin colonies of 1–2 mm in diameter after 7–10 days of incubation on YEM agar plates at 28 °C. The bacteria are able to grow at 15 °C–30 °C temperature, in 0.0–0.5% NaCl concentrations and in the pH range 5–10. The type strain ERR11^T^ and all other strains in the novel group were not able to grow at pH 4, at 4 °C and 35 °C, and in the 1–5% NaCl range (Additional file 1: Table S1). The carbon source utilization pattern of the type strain ERR11^T^ and other strains was tested as previously described [22] using Biolog GN2 plates with 95 carbon sources, following the manufacturer’s guideline [45]. Concisely, bacterial colonies grown on YEM agar were transferred and incubated on R2A media. Bacterial suspension was made by transferring colonies from R2A media into 0.5% (*w*/*v*) saline solution. Then, each of the wells of the Biolog GN2 Microplate was filled with 150 μl of the suspension. The results were recorded as positive when the wells turned purple after 4, 24, 48 h or 96 h incubation at 28 °C [46]. The carbon source utilization characteristics are presented in Additional file 2: Table S2. In general, the test strains showed a positive reaction for 66 of the carbon sources and negative reaction for 29 of the carbon sources (Additional file 2: Table S2). Despite that the diversity in carbon utilization patterns was minimal among test strains and between reference strain 10.1601/nm.1463 10.1601/strainfinder?urlappend=%3Fid%3DCCBAU+10071 ^T^ [37], only the test strains responded positively for adonitol, xylitol, and cis–aconitic acid carbon sources.

**Table 1:** Classification and general features of *Bradyrhizobium shewense* sp. nov. ERR11^T^ and *B. yuanmingense* CCBAU 10071^T^ [94]

MIGS ID	Property	ERR11^T^		CCBAU 10071^T^	
		Term	Evidence code	Term	Evidence code
		Domain *Bacteria*	TAS [95]	Domain *Bacteria*	TAS [95]
		Phylum 10.1601/nm.808	TAS [96]	Phylum 10.1601/nm.808	TAS [96]
		Class 10.1601/nm.809	TAS [97, 98]	Class 10.1601/nm.809	TAS [97, 98]
	Classification	Order 10.1601/nm.1277	TAS [98, 99]	Order 10.1601/nm.1277	TAS [98, 99]
		Family 10.1601/nm.1458	TAS [98, 100]	Family 10.1601/nm.1458	TAS [98, 100]
		Genus 10.1601/nm.1459	TAS [3]	*Genus* 10.1601/nm.1459	TAS [3]
		Species 10.1601/nm.30737 sp. nov.	IDA	Species 10.1601/nm.1463	TAS [37]
		Type strain ERR11^T^	IDA	Type strain CCBAU 10071^T^	TAS [37]
	Gram stain	Negative	IDA	Negative	IDA
	Cell shape	Rod	IDA	Rod	IDA
	Motility	Motile	IDA	Motile	IDA
	Sporulation	Non-sporulating	IDA	Non-sporulating	IDA
	Temperature range	Mesophile	IDA	Mesophile	TAS [37]
	Optimum temperature	28 °C	IDA	28 °C	TAS [37]
	pH range; Optimum	5–10; 7	IDA	6.5–7.5; 7	TAS [37]
	Carbon source	Varied (Additional file 2)	IDA	Varied	TAS [37]
MIGS-6	Habitat	Soil, root nodule	[4]	Soil, root nodule	TAS [37]
MIGS-6.3	Salinity	Non-halophile	IDA	Non-halophile	TAS [37]
MIGS-22	Oxygen requirement	Aerobic	IDA	Aerobic	TAS [37]
MIGS-15	Biotic relationship	Free living, symbiotic	IDA	Free living, symbiotic	TAS [37]
MIGS-14	Pathogenicity	Non-pathogenic	NAS	Non-pathogenic	NAS
MIGS-4	Geographic location	Central Ethiopia	[4]	Beijing, China	TAS [37]
MIGS-5	Sample collection	September, 2007	[4]	1995	TAS [37]
MIGS-4.1	Latitude	08^o^ 59' 38"	[4]	Not reported	TAS [37]
MIGS-4.2	Longitude	038^o^ 4' 18.5"	[4]	Not reported	TAS [37]
MIGS-4.4	Altitude	2327	[4]	Not reported	TAS [37]

Evidence codes – *IDA* Inferred from Direct Assay, *TAS* Traceable Author Statement (i.e., a direct report exists in the literature), *NAS* Non-traceable Author Statement (i.e.,not directly observed for the living, isolated sample, but based on a generally accepted property for the species, or anecdotal evidence). These evidence codes are from the Gene Ontology project [101]

Type strain 10.1601/strainfinder?urlappend=%3Fid%3DCCBAU+10071 ^T^ and other strains in 10.1601/nm.1463 were first described as distinct species using phenotypic features, SDS–PAGE analysis of whole–cell proteins, DNA–DNA hybridization and16S rRNA gene sequence analyses [37]. In agreement with the previous study, in this study based on *recA–glnII–rpoB* sequence analysis, the strains belonging to 10.1601/nm.1463 formed a district branch in Fig. 1. 10.1601/nm.1463 10.1601/strainfinder?urlappend=%3Fid%3DCCBAU+10071 ^T^ is motile and Gram–negative. The rod–shaped form (Fig. 2) has dimensions of approximately 0.5 μm in width and 1.5–2.0 μm in length. It is slow–growing, forming colonies with about 1–2 mm diameter after 7 days incubation at 28 °C on YMA. The optimum growth temperature reported was between 25 °C and 30 °C [37]. The organism grows best at pH 6.5–7.5 and growth recorded negative at pH 5.0 and pH 10.0, 10 °C or 40 °C and with 1.0% NaCl in YEMA [37]. Minimum Information about the Genome Sequence (MIGS) of 10.1601/strainfinder?urlappend=%3Fid%3DCCBAU+10071 ^T^ is provided in Table 1.

#### Symbiotaxonomy

The symbiotic properties of the strains in 10.1601/nm.30737 sp. nov. was studied in our previous study [5]. The strains recovered from nodules of *Indigofera* spp. [30] and *Crotalaria* spp. [29] formed an effective symbiotic association with the original host plants and also on soybean plants [5]. The type strain ERR11^T^ and other strains were again tested in this study for nodulation and nitrogen fixation ability on *E. brucei* [26], *Indigofera arrecta* [47] and *Crotalaria juncea* [48] as well as on food legumes soybean and peanut (*Arachis hypogaea*) [49]. All the sterilization and germination methods for *I. arrecta* [47] and *C. juncea* [48] seeds were as described previously [5]. Seeds from *E. brucei* [26], soybean and peanut were sterilized by soaking in 70% alcohol for 3 min and a sodium hypochlorite solution for 3 min followed by rinsing with 5–6 changes of sterilized water. *E. brucei* [26] seeds were germinated at room temperature (at about 25 °C) on 0.75% water agar or by wrapping with a sterilized paper towel. The soybean and peanut were germinated at 28 °C on 0.75% water agar. The symbiotic characteristics of 10.1601/nm.30737 sp. nov. strains are presented in Additional file 1: Table S1. The results show that the type strain ERR11^T^ and other strains obtained from *E. brucei* [26]*,* *Crotalaria* spp. [29] and *Indigofera* spp. [30] formed an effective symbiosis with *E. brucei* [26]*, I. arrecta* [47], soybean or peanut plants, suggesting the same origin of symbiotic genes among the rhizobia nodulating the legume plants *E. brucei, I. arrecta,* soybean or peanut. Only the strains from *Crotalaria* spp. [29] and *Indigofera* spp. [30] were able to form effective nodules on *C. juncea* plants [48]. Strains from *E. brucei* [26] including the type strain were unable to form effective symbiotic associations with soybean plants. 10.1601/nm.1463 is 10.1601/strainfinder?urlappend=%3Fid%3DCCBAU+10071 ^T^ was isolated from the nodules of the *Lespedeza cuneata* [50] legume in Beijing, China. In addition to its original host, the strain was also able to form an ineffective symbiotic association with *Medicago sativa* [51] and *Melilotus albus* [37, 52].

## Genome sequencing information

### Genome project history

Type strains ERR11^T^ and 10.1601/strainfinder?urlappend=%3Fid%3DCCBAU+10071 ^T^ were sequenced at the DOE–JGI as part of the Genomic Encyclopedia of Bacterial and Archaeal Type Strains, Phase III: the genomes of soil and plant–associated and newly described type strains sequencing project. The plant and soil associated bacteria were considered for sequencing to understand better their environmental and agricultural importance from the sequence information. The sequencing project was also designed to produce genome sequence data that can be used for bacterial classification studies and for a description of a new species using ANI and Genome–to–Genome–Distance values [36]. Based on our previous MLSA, the type strain ERR11^T^ together with other strains formed a distinctive phylogenetic group without including any known 10.1601/nm.1459 species, and this group representing most likely a new species [5]. Therefore, the aim of the genome sequencing of ERR11^T^ was to describe the group as a new species by comparing the genome sequence data of ERR11^T^ with the genome sequences of other 10.1601/nm.1459 species present in public databases. For this purpose, the type strain 10.1601/strainfinder?urlappend=%3Fid%3DCCBAU+10071 ^T^ [37] was also sequenced in this study to be used as a reference for our genome sequence comparison analysis. The ERR11^T^ genome project is deposited at the DOE–JGI genome portal [53] as well as at European Nucleotide Archive [54] under accession numbers FMAI01000001–FMAI01000102. The genome sequence of 10.1601/strainfinder?urlappend=%3Fid%3DCCBAU+10071 ^T^ is also available at DOE–JGI genome portal [53] and at European Nucleotide Archive [55] under accession numbers FMAE01000001–FMAE01000108. The Sequencing, assembling, finishing, and annotation were performed by the DOE–JGI [53]. The genome projects information is depicted in Table 2.

**Table 2:** Project information

MIGS ID	Property	Term, ERR11^T^	Term, CCBAU 10071^T^
MIGS 31	Finishing quality	High-quality draft	High-quality draft
MIGS-28	Libraries used	Illumina std. shotgun library	Illumina std. shotgun library
MIGS 29	Sequencing platforms	Illumina HiSeq 2500, Illumina HiSeq 2500–1 TB	Illumina HiSeq 2500, Illumina HiSeq 2500–1 TB
MIGS 31.2	Fold coverage	225.2X	279.9×
MIGS 30	Assemblers	Velvet (version 1.2.07), Allpaths–LG (version r46652)	Velvet (version 1.2.07), Allpaths–LG (version r46652)
MIGS 32	Gene calling method	Prodigal	Prodigal
	Locus Tag	ATF67	ATF66
	GenBank ID	FMAI01000000	FMAE01000000
	GenBank Date of Release	01-AUG-2016	01-10.1601/strainfinder?urlappend=%3Fid%3DAUG+2016
	GOLD ID	Gp0108279	Gp0108280
	BIOPROJECT	PRJNA303280	PRJNA303279
MIGS 13	Source Material Identifier	ERR11	CCBAU 10071
	Project relevance	Symbiotic N_2_ fixation, agriculture	Symbiotic N2 fixation, agriculture

### Growth conditions and genomic DNA preparation

The growth conditions and DNA isolation methods were as previously described [22]. In brief, the strains ERR11^T^ =10.1601/strainfinder?urlappend=%3Fid%3DHAMBI+3532 ^T^ and 10.1601/strainfinder?urlappend=%3Fid%3DCCBAU+10071 ^T^ = 10.1601/strainfinder?urlappend=%3Fid%3DLMG+21827 ^T^ were grown on YEM agar plates at 28 °C for 7–10 days and a pure colony of the cultures was transferred and grown in YEM broth till the culture reached late–logarithmic phase. The genomic DNAs were extracted from cell pellets following the CTAB DNA extraction protocol of the DOE–JGI [56].

### Genome sequencing and assembly

Strains ERR11^T^ and 10.1601/strainfinder?urlappend=%3Fid%3DCCBAU+10071 ^T^ were sequenced at the DOE–JGI by using the Illumina technology [57]. An Illumina std. shotgun library was constructed and sequenced using the Illumina HiSeq 2000 platform which produced 7,620,202 reads totaling 1150.7 Mb for ERR11^T^ and 9,923,442 reads counting 1498.4 Mb of 10.1601/strainfinder?urlappend=%3Fid%3DCCBAU+10071 ^T^. Details regarding the general aspects of library construction and sequencing methods can be found at the DOE–JGI website [53]. Artifacts from Illumina sequencing and library preparation were removed by passing all raw Illumina sequence data through DUK, filtering program developed by DOE–JGI [58]. The filtered Illumina reads were assembled first using Velvet (version 1.2.07) [59] and 1–3 kb simulated paired–end reads were created from Velvet contigs using wgsim (version 0.3.0) [60]. The Illumina reads were then assembled with the simulated read pairs using Allpaths–LG (version r46652) [61]. The final draft genome assembly comprises 9.2 Mb genome size containing 107 contigs in 102 scaffolds for strain ERR11^T^; 109 contigs in 108 scaffolds with a total size of 8.2 Mb for 10.1601/strainfinder?urlappend=%3Fid%3DCCBAU+10071 ^T^. The final assembly was based on 1399.7 Mb Illumina data and 225.2X input read coverage for the strain ERR11^T^; 1399.7 Mb Illumina data and 279.9× input read coverage for the strain 10.1601/strainfinder?urlappend=%3Fid%3DCCBAU+10071 ^T^.

### Genome annotation

Genes were first predicted by the Prodigal [62] program at the DOE–JGJ annotation pipeline [63], followed by a round of manual curation using GenePRIMP [64]. The predicted CDSs were translated and functionally annotated by searching against the NCBI non redundant database, UniProt, TIGRFam, Pfam, KEGG, COG, and InterPro databases. The tRNAScanSE tool [65] was used to identify tRNA genes and ribosomal RNA genes were predicted by searches against the ribosomal RNA genes in the SILVA database [66]. Other non–coding RNAs such as the RNA components of the protein secretion complex and the RNase P were identified by searching the genomes for the corresponding Rfam profiles using INFERNAL [67]. Additional gene prediction and functional annotation of the predicted genes were accomplished by using the Integrated Microbial Genomes (IMG) platform [68] developed by DOE–JGI [69].

## Genome properties

The genome of ERR11^T^ consists 102 scaffolds with a total size of 9,163,226 bp and a 63.2% G + C content. From a total 8634 genes, 8548 were protein–coding genes and 86 RNA encoding genes. The genome of 10.1601/strainfinder?urlappend=%3Fid%3DCCBAU+10071 ^T^ is arranged in 108 scaffolds and has a size of 6,928,453 bp with a 63.8% G + C content and of the 7861 predicted genes 7776 were protein–coding genes and 85 were RNAs–coding genes (Table 3). The majority of the protein–coding genes of ERR11^T^ (72.8%) and 10.1601/strainfinder?urlappend=%3Fid%3DCCBAU+10071 ^T^ (72.6%) were annotated to functions and the remaining 2266 (26.3%) and 2073 (26.4%) genes were without a functional prediction for ERR11^T^ and 10.1601/strainfinder?urlappend=%3Fid%3DCCBAU+10071 ^T^, respectively. About 62% CDSs of ERR11^T^ and 63% CDSs of 10.1601/strainfinder?urlappend=%3Fid%3DCCBAU+10071 ^T^ were assigned to COG functional categories. The distribution of the genes assigned into COGs functional categories is presented in Table 4.

**Table 3:** Genome statistics

Attribute	ERR11^T^		CCBAU 10071^T^	
	Value	% of Total	Value	% of Total
Genome size (bp)	9,163,226	100%	8,201,522	100%
DNA coding (bp)	8548	99%	6,928,453	84.48%
DNA G + C (bp)	5,792,812	63.22%	5,230,108	63.77%
DNA scaffolds	102	100%	108	100%
Total genes	8634	100%	7861	100%
Protein coding genes	8548	99%	7776	98.92%
RNA genes	86	1%	85	1.08%
Pseudo genes	not determined		not determined	
Genes in internal clusters	1889	21.88%	1457	18.53%
Genes with function prediction	6282	72.76%	5703	72.55%
Genes assigned to COGs	5346	61.92%	4913	62.50%
Genes with Pfam domains	6555	75.92%	6014	76.50%
Genes with signal peptides	924	10.70%	812	10.33%
Genes with transmembrane helices	1956	22.65%	1772	22.54%
CRISPR repeats	3		1

**Table 4:** Number of genes associated with general COG functional categories

Code	ERR11^T^		CCBAU 10071^T^		Description
	Value	%age	Value	%age	
J	225	3.65%	231	4.08%	Translation, ribosomal structure and biogenesis
A	0	00%	0	00%	RNA processing and modification
K	458	7.44%	392	6.93%	Transcription
L	135	2.19%	143	2.53%	Replication, recombination and repair
B	2	0.03%	2	0.04%	Chromatin structure and dynamics
D	36	0.58%	39	0.69%	Cell cycle control, Cell division, chromosome partitioning
V	162	2.63%	134	2.37%	Defense mechanisms
T	288	4.68%	263	4.47%	Signal transduction mechanisms
M	316	5.13%	300	5.3%	Cell wall/membrane biogenesis
N	106	1.72%	109	1.93%	Cell motility
U	85	1.38%	113	2%	Intracellular trafficking and secretion
O	245	3.98%	221	3.9%	Posttranslational modification, protein turnover, chaperones
C	440	7.15%	378	6.68%	Energy production and conversion
G	438	7.11%	339	5.97%	Carbohydrate transport and metabolism
E	665	10.8%	623	11.01%	Amino acid transport and metabolism
F	98	1.59%	94	1.66%	Nucleotide transport and metabolism
H	309	5.02%	271	4.79%	Coenzyme transport and metabolism
I	413	6.71%	398	7.03%	Lipid transport and metabolism
P	358	5.81%	311	5.49%	Inorganic ion transport and metabolism
Q	266	4.32%	278	4.91%	Secondary metabolites biosynthesis, transport and catabolism
R	684	11.11%	626	11.06%	General function prediction only
S	353	5.73%	333	5.83%	Function unknown
–	3288	38.08%	2948	37.5%	Not in COGs

The total is based on the total number of protein coding genes in the genome

## Insights from the genome sequence

### Genome wide comparative analysis

The strains belonging to 10.1601/nm.30737 sp. nov. formed their own group close to 10.1601/nm.25806 branch on the phylogenetic tree reconstructed based on *recA–glnII–rpoB* concatenated gene sequences (Fig. 1). Comparative analysis of the genome sequences between type strain ERR11^T^ and relatively close references was thus done for detail taxonomic study of the unique group and to describe it as a novel species. Among the reference genomes presented in the Fig. 1, completely sequenced 10.1601/nm.1460 10.1601/strainfinder?urlappend=%3Fid%3DUSDA+6 ^T^ [3, 70] and 10.1601/nm.24498 10.1601/strainfinder?urlappend=%3Fid%3DUSDA+110 ^T^ [71, 72] and draft sequences of 10.1601/nm.27386 26795^T^ [42, 43], 10.1601/nm.1460 10.1601/strainfinder?urlappend=%3Fid%3DUSDA+4 [73], 10.1601/nm.1459 *sp.* 10.1601/strainfinder?urlappend=%3Fid%3DCCBAU+15615 [74], 10.1601/nm.1462 strains (10.1601/strainfinder?urlappend=%3Fid%3DCCBAU+83689, 10.1601/strainfinder?urlappend=%3Fid%3DCCBAU+05525) [74], 10.1601/nm.1463 strains (10.1601/strainfinder?urlappend=%3Fid%3DCGMCC+1.3531, 10.1601/strainfinder?urlappend=%3Fid%3DCCBAU+35157) [37, 74], 10.1601/nm.23808 10.1601/strainfinder?urlappend=%3Fid%3DCGMCC+1.10947 ^T^ [41], 10.1601/nm.23259 10.1601/strainfinder?urlappend=%3Fid%3DCCBAU+23303 ^T^ [75] and 10.1601/nm.25639 BR3351^T^ [76] were collected from the DOE–JGI genome portal [53] as well as from the GenBank database [77]. The type strain 10.1601/nm.1463 10.1601/strainfinder?urlappend=%3Fid%3DCCBAU+10071 ^T^ [37] sequenced in this study was also included in the comparative analyses.

To evaluate the similarity between the genomes, we calculated genome–wide ANI by averaging the nucleotide identity of orthologous genes identified as bidirectional best hits as previously described [22, 24]. Based on this method, 96.5% ANI and 0.6 AF were set as the threshold values between strains in the same species [24]. In addition, DDH values were predicted between the genomes by using Genome–to–Genome Distance Calculator (GGDC) [78, 79]. This program computes the distance between genomes using three different formals: 1, high–scoring segment pairs (HSPs) /total length; 2, identities /HSP length; 3, identities/total length. The formula 2 proved to be a robust and recommended method for draft genome distance comparison [80].

The ANI values and DDH estimated results are presented in Table 5. The soybean–nodulating strain 10.1601/strainfinder?urlappend=%3Fid%3DUSDA+4 was previously classified as 10.1601/nm.1460 10.1601/strainfinder?urlappend=%3Fid%3DUSDA+4 based on sequence analysis of 16S rRNA gene and the internally transcribed spacer region of the 5′–23S rRNA gene [73]. However, the ANI value between type the strain 10.1601/nm.1460 10.1601/strainfinder?urlappend=%3Fid%3DUSDA+6 ^T^ and USD 4 was 90.2% and the DDH value between the two was 39.0%, suggesting that 10.1601/strainfinder?urlappend=%3Fid%3DUSDA+4 does not belong to the 10.1601/nm.1460 species. The strains 10.1601/strainfinder?urlappend=%3Fid%3DUSDA+4 and 10.1601/strainfinder?urlappend=%3Fid%3DCCBAU+15615 were tightly grouped with strains in 10.1601/nm.25806 on the phylogenetic tree in Fig. 1. Both 10.1601/strainfinder?urlappend=%3Fid%3DUSDA+4 and 10.1601/strainfinder?urlappend=%3Fid%3DCCBAU+15615 shared 99% *recA–glnII–rpoB* sequence identity with 10.1601/nm.25806 OO99^T^ and 10.1601/nm.25806 OO100 [39]. Even though the reference strains OO99^T^ and OO100 were not sequenced and not included in our ANI calculation, the *recA–glnII–rpoB* sequence analysis result strongly indicates that both 10.1601/strainfinder?urlappend=%3Fid%3DUSDA+4 and 10.1601/strainfinder?urlappend=%3Fid%3DCCBAU+15615 belong to 10.1601/nm.25806. The ANI values between type strain ERR11^T^ and references ranged from 86.8 to 95.2%, which is below 96.5%, the value for strains belong to the same species [24]. The closest strains were 10.1601/strainfinder?urlappend=%3Fid%3DUSDA+4 and 10.1601/strainfinder?urlappend=%3Fid%3DCCBAU+15615, with 95.2% ANI values followed by 10.1601/nm.1462 10.1601/strainfinder?urlappend=%3Fid%3DCCBAU+83689 and 10.1601/strainfinder?urlappend=%3Fid%3DCCBAU+05525, both sharing 89.6% ANI with strain ERR11^T^. The DDH predicted between strain ERR11^T^ and references were in the range of 31.1^_^58.5%. The highest DDH value obtained between ERR11^T^ and 10.1601/strainfinder?urlappend=%3Fid%3DCCBAU+15615 (58.5%) followed by 10.1601/strainfinder?urlappend=%3Fid%3DUSDA+4 (53.1%) is below the threshold of 70%, which is commonly used value for species delineation [78, 79]. In agreement with *recA–glnII–rpoB* gene sequence analysis, both ANI and DDH results revealed that the closest strains for ERR11^T^ were strains belong to 10.1601/nm.25806 group (10.1601/strainfinder?urlappend=%3Fid%3DCCBAU+15615 and 10.1601/strainfinder?urlappend=%3Fid%3DUSDA+4). Nevertheless, both the ANI and DDH values between ERR11^T^ and 10.1601/strainfinder?urlappend=%3Fid%3DCCBAU+15615 or 10.1601/strainfinder?urlappend=%3Fid%3DUSDA+4 were below the cutoff values of the strains of the same species, suggesting that ERR11^T^ belong to the novel group.

**Table 5:** ANI and DDH Genomic comparison between 10.1601/nm.30737 sp. nov. ERR11^T^ and reference 10.1601/nm.1459 species

	Genome name	NCBI/ENA accession number	MSLA	ANI was computed from protein-coding genes of the genomes using the MiSI program													
			1	1	2	3	4	5	6	7	8	9	10	11	12	13	14
1	*B. shewense* sp.nov. ERR11^T^	FMAI01000000			95.2	95.2	89.6	89.3	89.3	89.2	89.1	89.0	89.0	89.0	89.0	86.9	89.6
2	*B. ottawaense* USDA 4	AXAF00000000	96.0	53.1		99.9	90.0	90.3	90.3	89.1	90.2	89.1	89.1	90.0	89.1	87.1	90.1
3	*B. ottawaense* CCBAU15615	AJQG0100000	96.0	58.3	99.0		90.2	90.3	90.4	89.2	90.3	89.2	89.1	90.0	89.2	87.1	90.3
4	*B. liaoningense* CCBAU 83689	AJQD00000000	95.0	36.6	38.0	38.7		89.7	89.6	88.8	89.2	90.4	90.4	89.6	90.4	87.0	99.9
5	*B. huanghuaihaiense* CCBAU 23303^T^	PRJNA255602	94.0	35.6	39.2	39.4	37.4		91.2	90.0	90.3	89.3	89.2	89.8	89.2	87.6	89.7
6	*B. diazoefficiens* USDA 110^T^	CP011360	94.0	35.7	39.3	39.6	37.2	42.1		89.4	91.0	88.8	88.8	89.7	88.8	87.7	89.6
7	*B.arachidis* LMG 26795^T^	FPBQ01000000	94.0	35.4	35.1	35.2	34.8	37.3	35.9		89.5	88.6	88.6	89.6	88.6	87.7	88.8
8	*B.japonicum* USDA 6^T^	AP012206	94.0	35.4	39.0	39.3	36.3	39.3	41.0	36.4		88.4	88.4	89.3	88.5	87.5	89.2
9	*B.yuanmingense* CCBAU 10071^T^	FMAE00000000	94.0	34.7	34.7	35.1	38.3	35.3	34.5	34.0	33.6		100.0	89.1	98.2	86.8	90.3
10	*B.yuanmingense* CGMCC1.3531	PRJNA255601	94.0	34.7	34.7	35.0	38.3	35.5	34.5	33.8	33.6	100.0		90.0	98.2	86.8	90.3
11	*B.daqingense* CGMCC 1.10947^T^	PRJNA255603	94.0	34.9	38.3	38.5	37.0	37.8	37.7	34.0	36.7	35.1	35.1		89.1	86.8	89.7
12	*B.yuanmingense* CCBAU 35157	AJQL00000000	94.0	34.7	35.0	35.1	38.4	35.5	34.5	34.0	33.7	82.5	82.5	35.0		86.8	90.4
13	*B. manausense* BR3351^T^	LJYG00000000	94.0	31.1	58.1	31.6	31.5	32.4	32.3	32.5	32.2	31.1	31.1	30.9	31.0		87.0
14	*B.liaoningense* CCBAU 05525	AJQC00000000	95.0	36.6	38.3	39.1	99.5	37.7	37.6	34.8	36.7	38.2	38.2	37.3	38.3	31.6	
			DDH values were predicted by the Genome-to-Genome Distance calculator 2.0, formula 2														

The numbers in MLSA column indicate *recA, glnII, rpoB* concatenated gene sequence similarities between ERR11^T^ and reference strains. The numbers below the diagonal are DDH values predicted between pairwise genomes. The numbers above the diagonal are ANI values between genomes; in all ANI calculations AF was > = 60%. Reference type strains are indicated with superscript ‘T’; B, *Bradyrhizobium*

**Fig. 3:**
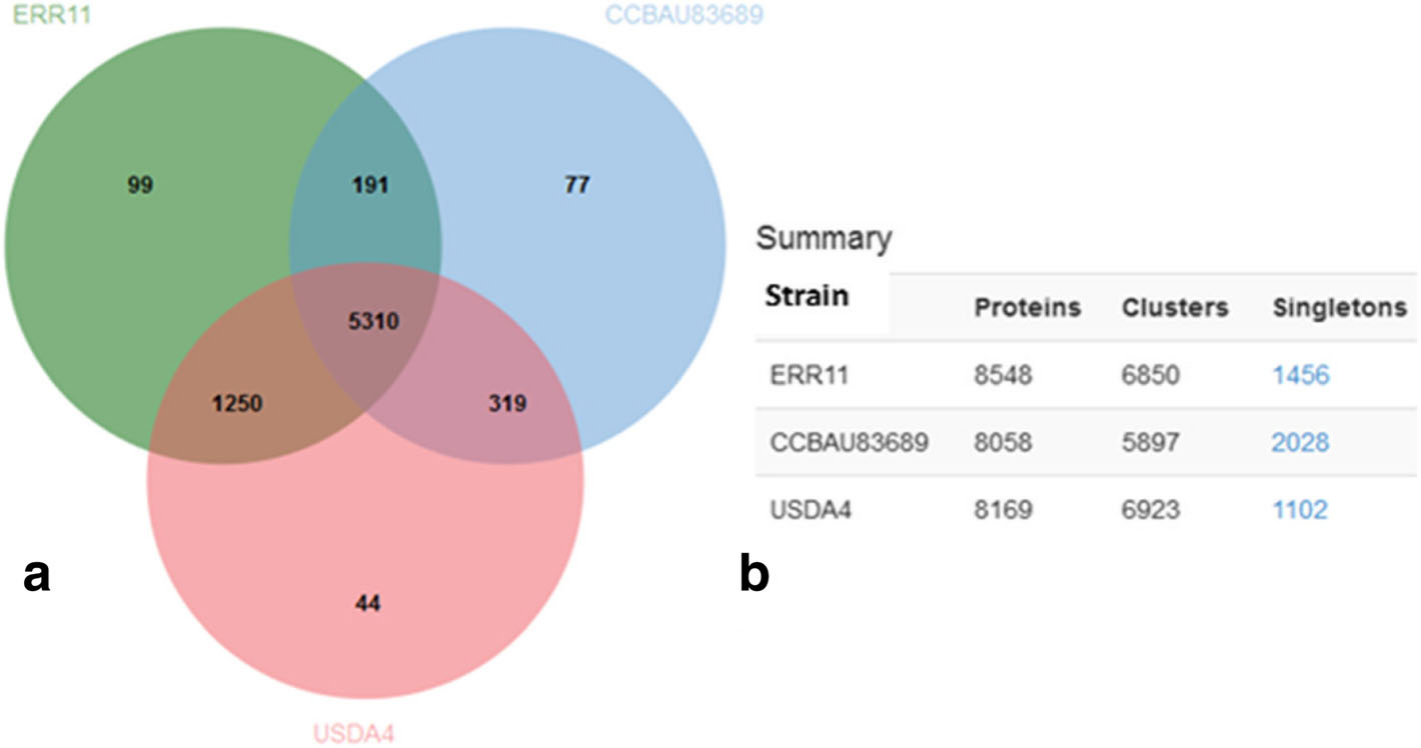
Venn diagram (panel **a**) plotted by OrthoVenn program shows shared orthologous protein clusters between three genomes (in the center): 10.1601/nm.30737 sp.nov. ERR11^T^, 10.1601/nm.25806 10.1601/strainfinder?urlappend=%3Fid%3DUSDA+4 and 10.1601/nm.1462 10.1601/strainfinder?urlappend=%3Fid%3DCCBAU+83689. The total number of protein sequences, the number of protein clusters comprising multiple protein families and also the number of singletons i.e. protein with no orthologous are summarized in (panel **b**) for each genome

Shared orthologous protein clusters between the genomes of ERR11^T^ and the closest reference strains 10.1601/strainfinder?urlappend=%3Fid%3DUSDA+4 and 10.1601/strainfinder?urlappend=%3Fid%3DCCBAU+83689 were identified using an OrthoVenn program [81] as described previously [22]. The orthologous clusters are shown in a Venn diagram (Fig. 3). The number of protein clusters identified in each of ERR11^T^, 10.1601/strainfinder?urlappend=%3Fid%3DUSDA+4 and 10.1601/strainfinder?urlappend=%3Fid%3DCCBAU+83689 was 6850, 5897 and 6923, respectively. In the genome of ERR11^T^, 99 of the clusters were identified as unique protein clusters without homologs in the other genomes. In 10.1601/strainfinder?urlappend=%3Fid%3DUSDA+4 and 10.1601/strainfinder?urlappend=%3Fid%3DCCBAU+83689, 44 and 77 protein clusters respectively, were also identified as unique clusters with no detectable homologous with other genomes. Of the total proteins used in the analysis 1456, 2028, 1102 were single copy gene clusters in ERR11^T^, 10.1601/strainfinder?urlappend=%3Fid%3DUSDA+4 and 10.1601/strainfinder?urlappend=%3Fid%3DCCBAU+83689, respectively. Of the clusters, in total 5310 homologous protein clusters were shared in common by all of the three genomes. Strain ERR11^T^ shares about 76.7% (6560) of its proteins with 10.1601/strainfinder?urlappend=%3Fid%3DUSDA+4 and 64.4% (5501) clusters with 10.1601/strainfinder?urlappend=%3Fid%3DCCBAU+83689. Based on the pairwise comparison, ERR11^T^ shared the highest number with strain 10.1601/strainfinder?urlappend=%3Fid%3DUSDA+4 with 1250 protein clusters and ERR11^T^ shared only 191 protein clusters with 10.1601/strainfinder?urlappend=%3Fid%3DCCBAU+83689. This result is in accordance with the phylogenetic tree (Fig. 1), ANI and DDH results (Table 5), supporting that strain 10.1601/strainfinder?urlappend=%3Fid%3DUSDA+4 (in the 10.1601/nm.25806 species group) is more closely related to ERR11^T^ compared to strain 10.1601/strainfinder?urlappend=%3Fid%3DCCBAU+83689 (in 10.1601/nm.1462).

### Comparative analysis of genes linked to symbiosis and denitrification

#### Symbiotic genes

**Fig. 4:**
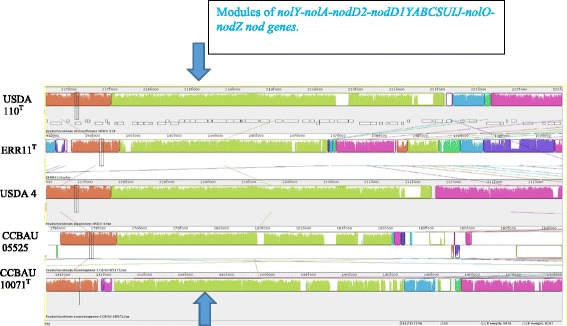
Mauve alignment comparing the genome of ERR11^T^ with the genome of 10.1601/strainfinder?urlappend=%3Fid%3DUSDA+110 ^T^, 10.1601/strainfinder?urlappend=%3Fid%3DUSDA+4, 10.1601/strainfinder?urlappend=%3Fid%3DCCBAU+05525 and CCBAU 10071^T^. The nod genes: *nolY-nolA-nodD2-nodD1YABCSUIJ-nolO-nodZ* indicated by the arrows are homologous and organized similarly between the genomes

The nodulation genes (*nod, nol, noe*) for the synthesis of the backbone of LCO Nod factors and substituent groups and genes coding for nitrogen fixation (*nif, fix*) are required in rhizobia–legume symbiosis [70, 72]. In order to search the symbiotic genes in ERR11^T^ and 10.1601/strainfinder?urlappend=%3Fid%3DCCBAU+10071 ^T^, the genomes were assembled against completely sequenced 10.1601/strainfinder?urlappend=%3Fid%3DUSDA+110 ^T^ and 10.1601/strainfinder?urlappend=%3Fid%3DUSDA+6 ^T^ using the Genome Gene Best Homologs package from program IMG–ER [69]. In addition, the symbiotic genes were also compared against other draft 10.1601/nm.1459 genomes: 10.1601/strainfinder?urlappend=%3Fid%3DLMG+26795 ^T^, 10.1601/strainfinder?urlappend=%3Fid%3DCGMCC+1.10947 ^T^, 10.1601/strainfinder?urlappend=%3Fid%3DCGMCC+1.10948 ^T^, 10.1601/strainfinder?urlappend=%3Fid%3DUSDA+4, and 10.1601/strainfinder?urlappend=%3Fid%3DCCBAU+05525. To see the arrangement of symbiotic genes, the genome of ERR11^T^ and references were aligned using the progressive Mauve alignment method [82]. Summary of the symbiotic genes identified in ERR11^T^ and 10.1601/strainfinder?urlappend=%3Fid%3DCCBAU+10071 ^T^ and their locations in the genomes and resemblance with genes in the reference genomes are shown in Additional file 3: Table S3. The main nodulation genes; *nolY–nolA–nodD2–nodD1YABCSUIJ–nolO–nodZ* were identified in scaffolds Ga0061098_1039 and Ga0061099_1014, in the genome of ERR11^T^ and 10.1601/strainfinder?urlappend=%3Fid%3DCCBAU+10071 ^T^, respectively. The result of the Mauve alignment (Fig. 4) shows that these genes are homologous and organized in the same region (module) similarly as the genes found in the genome of 10.1601/strainfinder?urlappend=%3Fid%3DUSDA+110 ^T^, 10.1601/strainfinder?urlappend=%3Fid%3DUSDA+4, and 10.1601/strainfinder?urlappend=%3Fid%3DCCBAU+05525. Additional nodulation genes of ERR11^T^ are scattered in scaffolds Ga0061098_1005 (*nodWV, nodM, noeL, nolXWTUV*), Ga0061098_1016 (*nodU*), Ga0061098_1006 (*nodT*) and Ga0061098_1031 (*noeE, noeI*). These genes are also identified in the genome of 10.1601/strainfinder?urlappend=%3Fid%3DCCBAU+10071 ^T^ in Ga0061099_1013 and Ga0061099_1018, Ga0061099_1014, Ga0061099_1005 and Ga0061099_1022, respectively.

In the genome of ERR11^T^, the genes coding for the nitrogen–fixing nitrogenase complex [83] are mainly located in scaffolds Ga0061098_1005 (*nifDKENX–nifT–nifB–nifZ–nifHQV–fixBCX*), and Ga0061098_1039 (*fixR–nifA–fixA*). The *nif/fix* genes in the genome of 10.1601/strainfinder?urlappend=%3Fid%3DCCBAU+10071 ^T^ are distributed in scaffolds Ga0061099_1013 (*nifDKENX*), Ga0061099_1041 (*nifT–nifB–nifZ*), Ga0061099_1036 (*nifHQV–fixBCX*), and Ga0061099_1014 (*fixR–nifA–fixA*). The *fix* genes (*fixK2–fixJL–fixNOPGHIS*), which are required for creating microoxic respiration for the rhizobia during symbiosis, are also conserved in the genomes of ERR11^T^ (in scaffold Ga0061098_1024) and 10.1601/strainfinder?urlappend=%3Fid%3DCCBAU+10071 ^T^ (in scaffold Ga0061099_10014) in a similar fashion as the homologous genes found in 10.1601/strainfinder?urlappend=%3Fid%3DUSDA+110 ^T^. Generally, the nodulation and nitrogen fixation genes of ERR11^T^ and 10.1601/strainfinder?urlappend=%3Fid%3DCCBAU+10071 ^T^ showed 70.0–100% sequence similarity with homologous genes found in the reference genomes of 10.1601/strainfinder?urlappend=%3Fid%3DUSDA+110 ^T^, 10.1601/strainfinder?urlappend=%3Fid%3DUSDA+6 ^T^, 10.1601/strainfinder?urlappend=%3Fid%3DUSDA+4, 10.1601/strainfinder?urlappend=%3Fid%3DCCBAU+23303 ^T^, 10.1601/strainfinder?urlappend=%3Fid%3DCGMCC+1.10947 ^T^, and 10.1601/strainfinder?urlappend=%3Fid%3DCCBAU+05525 (Additional file 3: Table S3). The nodulation and nitrogen fixation genes of ERR11^T^ mostly showed the highest sequence similarities (>90%) specifically with homologous genes found in the genome of peanut–nodulating strain 10.1601/strainfinder?urlappend=%3Fid%3DLMG+26795 ^T^, suggesting that these strains may have a similar origin of symbiotic genes.

Nitrogen fixation in symbiosis is an ATP–dependent energy intensive reaction, where energy is released in the form of H_2_ as a result of the reduction of N_2_ by nitrogenase. The rhizobia which have hydrogen–uptake systems are capable of recycling the released H_2_ in the rhizobia–legume symbiosis [84]. This way some rhizobia increase the energy efficiency in symbiosis and consequently the nitrogen–fixation and legume productivity. The hydrogenase uptake complex is coded by clusters of *hupNCUVSLCDFGHIJK*, *hypABFCDE*, and *hoxXA* genes [70, 72, 85, 86]*.* Clusters of *hupSLCFHK* and *hypBFDE* genes were identified in the genomes of ERR11^T^ in scaffold Ga0061098_1005 and in 10.1601/strainfinder?urlappend=%3Fid%3DCCBAU+10071 ^T^ in scaffold Ga0061099_1013 (Additional file 3: Table S3). The composition of hydrogenase genes in the clusters *hup, hyp* and *hox* and their expression can be different between rhizobial species and are also missing in some rhizobia [70, 72, 84]. Rhizobia with the functional hydrogenase uptake system, such as strain 10.1601/strainfinder?urlappend=%3Fid%3DUSDA+110 ^T^ contained a complete set of *hup–hyp–hox* genes [72]. In the genomes of ERR11^T^ and 10.1601/strainfinder?urlappend=%3Fid%3DCCBAU+10071 ^T^, some of the genes are missing or incomplete. Therefore, further study and complete sequencing may confirm if the hydrogenase uptake system is functional in these strains.

#### Denitrifying genes

Denitrification is a process by which NO_3_^−^ and NO_2_^−^ are reduced to N_2_ when NO_3_^−^ or NO_2_^−^ is used by microorganisms as a final electron acceptor for respiration as an alternative in oxygen limitation. NO and N_2_O are produced as intermediate products during this process [87]. Thus, denitrification result in nitrogen losses from terrestrial and aquatic ecosystems and also contribute to the production of a potent greenhouse gas, N_2_O. The denitrification is common among the bacteria in the 10.1601/nm.808 class and also in *Archaea* [88]. Symbiotic nitrogen–fixing rhizobia, particularly species belonging to 10.1601/nm.1459 were reported to be involved in the denitrification process in low oxygen environments [89]. All or some of the genes for NO_3_^−^, NO_2_^−^, NO and N_2_O reductions were found in several rhizobial species investigated thus far [90] and emission of N_2_O by symbiotic rhizobia inside the root nodules was reported [91, 92]. Nitrogen–fixing 10.1601/strainfinder?urlappend=%3Fid%3DUSDA+110 ^T^ is known to denitrify as free living and also in the symbiotic condition in root nodules of soybean [89, 93]. Strain 10.1601/strainfinder?urlappend=%3Fid%3DUSDA+110 ^T^ requires *napEDABC, nirK, norCBQD,* and *nosRZDFYLX* gene clusters for NO_3_^−^, NO_2_^−^, NO and N_2_O reductase, respectively [90]. In the genome of ERR11^T^, the *napCBADE, norDQBCE, nosRZDYL* cluster of genes are present in the scaffolds Ga0061098_1005, Ga0061098_1001, and Ga0061098_1006, respectively. The gene for nitrite reductase (*nir*) was not found in ERR11^T^. Therefore, the nitrite reductase activity may be lacking in ERR11^T^ and denitrification in this strain may depend only on nitrate, nitric oxide, and nitrous oxide reductase reactions. The genome of 10.1601/strainfinder?urlappend=%3Fid%3DCCBAU+10071 ^T^ harbors only denitrifying genes *napEDABC* and *norCBQD* for nitrate and nitric oxide reduction, respectively (Additional file 3: Table S3). Further experimental study with appropriate methods and techniques can help to understand better the presence of denitrification enzyme activities in the type strains ERR11^T^ and 10.1601/strainfinder?urlappend=%3Fid%3DCCBAU+10071 ^T^ and to confirm if the type strains are involved in the denitrification process and N_2_O emission.

## Conclusion

In this study, we present the genome sequences of 10.1601/nm.30737 sp. nov. strain ERR11^T^ and the type strain 10.1601/nm.1463 10.1601/strainfinder?urlappend=%3Fid%3DCCBAU+10071 ^T^. The draft genome size of ERR11^T^ and 10.1601/strainfinder?urlappend=%3Fid%3DCCBAU+10071 ^T^ is about 9.2Mbp and 8.2Mbp, respectively. Type strain 10.1601/strainfinder?urlappend=%3Fid%3DCCBAU+10071 ^T^ was selected for sequencing to be used as a reference for our comparative genomic analysis. The genomes of the type strains ERR11^T^ and 10.1601/strainfinder?urlappend=%3Fid%3DCCBAU+10071 ^T^ carry genes for nodulation, nitrogen fixation, the hydrogen–uptake system as well as genes for denitrification. The *nod* genes *nolY–nolA–nodD2–nodD1YABCSUIJ–nolO–nodZ* in the genomes of ERR11^T^ and 10.1601/strainfinder?urlappend=%3Fid%3DCCBAU+10071 ^T^ are organized similarly as homologous genes identified in the genomes of 10.1601/strainfinder?urlappend=%3Fid%3DUSDA+110 ^T^, 10.1601/strainfinder?urlappend=%3Fid%3DUSDA+4, and 10.1601/strainfinder?urlappend=%3Fid%3DCCBAU+05525. The nodulation and nitrogen fixation genes of ERR11^T^ share high sequence similarity with peanut–nodulating type strain 10.1601/nm.27386 10.1601/strainfinder?urlappend=%3Fid%3DLMG+26795 ^T^ [42, 43] The denitrification genes *nap, nor* and *nos* of ERR11^T^ and *nap* and *nor* of 10.1601/strainfinder?urlappend=%3Fid%3DCCBAU+10071 ^T^ are homologous to the genes in found in the genome of 10.1601/strainfinder?urlappend=%3Fid%3DUSDA+110 ^T^, a known denitrifying rhizobium, indicating that ERR11^T^ and 10.1601/strainfinder?urlappend=%3Fid%3DCCBAU+10071 ^T^ may involve in reduction of nitrate, nitric oxide, or nitrous oxide. Based on the phylogenetic analyses of *recA–glnII–rpoB* sequences, the strains (ERR2A, ERR2B, ERR11, ERR13, 10.1601/strainfinder?urlappend=%3Fid%3DCIR+42, 10.1601/strainfinder?urlappend=%3Fid%3DCSR+10B, IAR8 and AURI6) belonging to the novel species formed a unique group within the genus 10.1601/nm.1459 *.* In order to verdict this result, comparative genomic analyses based on ANI calculation and DDH methods were done. The results from both ANI and DDH supported the result from the phylogenetic analysis, in which the genome of the type strain ERR11^T^ showed 95.2% ANI and 53.1 DDH similarity with the closest reference strain 10.1601/strainfinder?urlappend=%3Fid%3DUSDA+4. These values are lower than the 96.5% ANI and 70% DDH cutoff values designed for strains of the same species. These results confirm that 10.1601/nm.30737 sp. nov. should be considered as a new 10.1601/nm.1459 species. Therefore, based on the phylogenetic analysis, ANI and DDH results and by including phenotypic characteristics, we formally propose the creation of 10.1601/nm.30737 sp. nov. that contains the strain ERR11^T^ (= 10.1601/strainfinder?urlappend=%3Fid%3DHAMBI+3532 ^T^=10.1601/strainfinder?urlappend=%3Fid%3DLMG+30162 ^T^). The type strain forms an effective nitrogen–fixing symbiosis with *E. brucei* [26]*, I. arrecta* (47) and peanut.

### Description of 10.1601/nm.30737 sp. nov.

10.1601/nm.30737 (she.wen’se. L. neut. Adj. *shewense* of Shewa, pertaining to Shewa, the region in Ethiopia, where the type strain was obtained). The bacteria are non–spore–forming, Gram–negative rods with a size of 1.0–2.4 μM in length and 0.7–1.0 μm width. Strains included in species are slow–growing, forming creamy, raised and smooth margin colonies of 1–2 mm in diameter after 7 to 10 days of incubation on YEMA plate containing Congo red at 28 °C and pH 7 optimal growth conditions. The strains are able to grow at 15 °C–30 °C, in 0.0–0.5 NaCl and at 5–10 pH ranges. They do not grow at pH 4, at 4 °C and at 35 °C and in 1–5% NaCl. In general, the type and the other strains in this species could ferment the following substrates as carbon sources in Biolog GN2 microplates; Tween 40, Tween 80, adonitol, L–arabinose, D–arabitol, glycogen, N–acetyl–D–glucosamine, adonitol, L–arabinose, D–arabitol, D–cellobiose, I–erythritol, D–fructose, L–fucose, D–galactose, α–D–glucose, α–D–lactose, lactulose, D–mannitol, D–mannose, D–melibiose, β–methyl–D–glucoside, D–raffinose, L–rhamnose, D–sorbitol, sucrose, turanose, xylitol, pyruvic acid methyl ester, acetic acid, succinic acid mono–methyl–ester, cis–aconitic acid, citric acid, formic acid, D–galactonic acid lactone, D–galacturonic acid, D–gluconic acid, D–glucosaminic acid, D–glucuronic acid, α–Hydroxybutyric acid, β–hydroxybutyric acid, γ–hydroxybutyric acid, p–hydroxy phenylacetic acid, itaconic acid, α–keto butyric acid, α–keto glutaric acid, α–keto valeric acid, D,L–lactic acid, propionic acid, quinic acid, D–saccharic acid, sebacic acid, succinic acid, bromosuccinic acid, succinamic acid, glucuronamide, L–alaninamide, D–alanine, L–alanine, L–alanyl–glycine, L–asparagine, L–aspartic Acid, L–glutamic acid, glycyl–L–aspartic acid, glycyl–L–glutamic acid, L–leucine, L–phenylalanine, L–proline, L–pyroglutamic acid, D–serine, L–threonine, urocanic acid, and glycerol. However, all the strains included in this test failed to oxidize α–cyclodextrin, glycogen, N–acetyl–D–galactosamine, N–acetyl–D–glucosamine, D–cellobiose, I–erythritol, gentiobiose, M–inositol, α–D–lactose, lactulose, D–melibiose, β–methyl–D–glucoside, D–raffinose, melonic acid, L–histidine, hydroxy–L–proline, L–ornithine, D,L–carnitine, γ–amino butyric acid, uridine, thymidine, phenyethyl–amine, putrescine, 2–aminoethanol, 2,3–butanediol, D,L–α–glycerol phosphate, α–D–glucose–1–phosphate, and D–glucose–6–phosphate. The type strain ERR11^T^ was obtained from root nodules of *E. brucei* [25] growing in Ethiopia. The genome size of the type strain is 9.2Mbp and the genome G + C content is 63.2%. The genome sequence of ERR11^T^ is available at the DOE–JGI genome portal [47] as well as at European Nucleotide Archive [48] from accession number FMAI01000001 to FMAI01000102. The type strain has been deposited in the HAMBI (10.1601/strainfinder?urlappend=%3Fid%3DHAMBI+3532 ^T^) and LMG (10.1601/strainfinder?urlappend=%3Fid%3DLMG+30162 ^T^) culture collections.

## Additional files

Additional file 1: Table S1. Phenotypic characteristics of *Bradyrhizobium shewense* sp. nov. strains. [102, 103] (DOCX 28 kb)

Additional file2:Table S2. Carbon sources utilization response between *Bradyrhizobium shewense* sp. nov. strains and reference type strain *B. yuanmingense* CCBAU 10071^T^. (DOCX 29 kb)

Additional file 3: Table S3. Symbiotic and denitrifying genes identified in the genomes of *Bradyrhizobium shewense*. sp. nov. ERR11^T^ and *B. yuanmingense* CCBAU 10071^T^. (XLSX 121 kb)

## Abbreviations

AF: Alignment fraction
ANI: Average nucleotide identity
CTAB: Cetyl Trimethyl Ammonium Bromide
DDH: DNA-DNA Hybridization
DOE: Department of Energy
GOLD: Genomes online database
H_2_: Dihydrogen
IMG: Integrated Microbial Genomes
IMG-ER: Integrated microbial genomes – expert review
JGI: Joint Genome Institute
LCO: Lipochito-Oligosaccharide
MIGS: Minimum information about a genome sequence
MiSI: Microbial species identifier
MLSA: Multilocus sequence analysis
N_2_: Dinitrogen
N_2_O: Nitrous oxide
NO: Nitric oxide
NO_2_: Nitrite
NO_3_: Nitrate
R2A: Reasoner’s 2A Agar
SDS-PAGE: Sodium dodecyl sulfate polyacrylamide gel electrophoresis
YEMA: Yeast Extract Mannitol Agara
