# Supplementary material for: Draft genome sequences of Bradyrhizobium shewense sp. nov. ERR11T and Bradyrhizobium yuanmingense CCBAU 10071T
Source: Stand Genomic Sci. 2017 Dec 5;12:74. doi: 10.1186/s40793-017-0283-x (PMC5717998; doi:10.1186/s40793-017-0283-x)
Supplement: Supplementary file 1 — Phenotypic characteristics of Bradyrhizobium shewense sp. nov. strains. [102, 103] (DOCX 28 kb) [file 40793_2017_283_MOESM1_ESM.docx]

Additional file 1 Table S1. Phenotypic characteristics of *Bradyrhizobium shewense* sp. nov. strains

|  | | | ERR11^T^ | ERR2A | ERR2B | ERR13 | CIR42 | CSR10B | AURI6 | IAR8 |
| --- | --- | --- | --- | --- | --- | --- | --- | --- | --- | --- |
| **Temperature** | | |  |  |  |  |  |  |  |  |
| 5^O^C (day 10-15) | | | - | - | - | - | - | - | - | - |
| 15^O^C (day 10-15) | | | **+** | **+** | **+** | + | **+** | **+** | **+** | **+** |
| 20 ^O^C (day10-15) | | | **+** | **+** | **+** | + | **+** | **+** | **+** | **+** |
| 28-30 ^O^C (7-10) | | | **+** | **+** | **+** | + | **+** | **+** | **+** | **+** |
| 35-36 ^O^C(day10-15) | | | - | - | - | - | - | - | - | - |
| **pH** | | |  |  |  |  |  |  |  |  |
| 4 (day10-15) | | | - | - | - | - | - | - | - | - |
| 5 (day7-10) | | | **+** | **+** | **+** | + | **+** | **+** | **+** | **+** |
| 7 (day 7-10) | | | **+** | **+** | **+** | + | **+** | **+** | **+** | **+** |
| 9 (day7-10) | | | **+** | **+** | **+** | + | **+** | **+** | **+** | **+** |
| 10 (day7-10) | | | **+** | **+** | **+** | + | **+** | **+** | **+** | **+** |
| **NaCl** | | |  |  |  |  |  |  |  |  |
| 0% (day7-10) | | | **+** | **+** | **+** | + | **+** | **+** | **+** | **+** |
| 0.5% (day7-10) | | | **+** | **+** | **+** | + | **+** | **+** | **+** | **+** |
| 1% (day10-15) | | | - | - | - | - | - | - | - | - |
| 2% (day10-15) | | | - | - | - | - | - | - | - | - |
| 3% (day10-15) | | | - | - | - | - | - | - | - | - |
| 5% (day 10-15) | | | - | - | - | - | - | - | - | - |
|  | | | | | | | | | | |
| **Host plant** | | | *E. brucei*  [26] | *E. brucei*  [26] | *E. brucei*  [26] | *E. brucei*  [26] | *C. incana*  [102] | *C. spinosa*  [103] | *Indig*  *ofera sp.*  [[30](https://plants.usda.gov/core/profile?symbol=INDIG)] | *I. arrecta*  [47] |
| **Nodulation Test** | |  | |  |  |  |  |  |  |  |
| ***E. brucei*** | nod | | **+** | **+** | **+** | ND | **+** | **-** | **+** | **+** |
|  | fix | | **+** | **+** | **+** | ND | **+** | **-** | **+** | **+** |
| ***C. juncea***  **[48]** | nod | | - | - | - | ND | + | + | + | + |
|  | fix | | - | - | - | ND | + | + | + | + |
| ***C. incana*** | nod | | ND | ND | ND | ND | + | + | ND | ND |
|  | fix | | ND | ND | ND | ND | + | + | ND | ND |
| ***I. arrecta*** | nod | | + | + | + | ND | + | + | + | + |
|  | fix | | + | + | + | ND | + | + | + | + |
| **Soybean** | nod | | - | + | - | ND | + | + | + | + |
|  | fix | | - | - |  | ND | - | - | + | - |
| **Peanut** | nod | | + | ND | ND | ND | ND | ND | + | ND |
|  | fix | | + | ND | ND | ND | ND | ND | + | ND |

Duration of incubation of the bacteria are indicated next to the pH, NaCl and temperature labels. +, growth; nod, nodule formation; fix, nitrogen fixation; -, no growth, no nodulation or no nitrogen fixation; ND, not determined, *E.; Erythrina, C.; crotalria, I.; Indigofera.*
