# Supplementary material for: Draft genome sequences of Bradyrhizobium shewense sp. nov. ERR11T and Bradyrhizobium yuanmingense CCBAU 10071T
Source: Stand Genomic Sci. 2017 Dec 5;12:74. doi: 10.1186/s40793-017-0283-x (PMC5717998; doi:10.1186/s40793-017-0283-x)
Supplement: Supplementary file 2 — Carbon sources utilization response between Bradyrhizobium shewense sp. nov. strains and reference type strain B. yuanmingense CCBAU 10071T. (DOCX 29 kb) [file 40793_2017_283_MOESM2_ESM.docx]

**Additional file2 Table S2.** Carbon sources utilization response between *Bradyrhizobium shewense* sp. nov. strains and reference type strain *B. yuanmingense* CCBAU 10071^T^

| **Carbon sources** | **IAR8** | **CSR10B** | **ERR11^T^** | **CIR42** | **AURI6** | **CCBAU 10071^T^** |
| --- | --- | --- | --- | --- | --- | --- |
| Water | - | - | - | - | - | - |
| α-Cyclodextrin | - | - | - | - | - | - |
| Dextrin | - | - | **+** | - | - | **+** |
| Glycogen | - | - | **-** | - | - | - |
| Tween 40 | **+** | **+** | **+** | **+** | **+** | **+** |
| Tween 80 | **+** | **+** | **+** | **+** | **+** | **+** |
| N-Acetyl-DGalactosamine | - | - | - | - | - | - |
| N-Acetyl-DGlucosamine | - | - | - | - | - | - |
| Adonitol | **+** | **+** | **+** | **+** | **+** | - |
| L-Arabinose | **+** | **+** | **+** | **+** | **+** | **+** |
| D-Arabitol | **+** | **+** | **+** | **+** | **+** | **+** |
| D-Cellobiose | - | - | - | - | - | - |
| i-Erythritol | - | - | **-** | - | - | - |
| D-Fructose | **+** | **+** | **+** | **+** | **+** | **+** |
| L-Fucose | **+** | **+** | **+** | **+** | **+** | **+** |
| D-Galactose | **+** | **+** | **+** | **+** | **+** | **+** |
| Gentiobiose | - | - | - | - | - | - |
| α-D-Glucose | **+** | **+** | **+** | **+** | **+** | **+** |
| m-Inositol | - | - | - | - | - | - |
| α-D-Lactose | - | - | - | - | - | - |
| Lactulose | - | - | **-** | - | - | - |
| Maltose | - | - | **+** | - | - | - |
| D-Mannitol | **+** | **+** | **+** | + | **+** | **+** |
| D-Mannose | **+** | **+** | **+** | **+** | **+** | **+** |
| D-Melibiose | - | - | - | - | - | - |
| β-Methyl-D-Glucoside | - | - | - | - | - | - |
| D-Psicose | - | t | **+** | - | - | **+** |
| D-Raffinose | - | - | - | - | - | - |
| L-Rhamnose | - | - | **+** | **+** | **+** | **+** |
| D-Sorbitol | **+** | - | **+** | - | **+** | **+** |
| Sucrose | - | - | **-** | - | - | - |
| D-Trehalose | - | + | **-** | - | - | - |
| Turanose | - | - | **+** | **+** | **+** | **+** |
| Xylitol | **+** | **+** | **+** | **+** | **+** | - |
| Pyruvic Acid Methyl Ester | **+** | **+** | **+** | **+** | **+** | **+** |
| Succinic Acid Mono-Methyl-Ester | **+** | **+** | **+** | **+** | **+** | **+** |
| Acetic acid | **+** | **+** | **+** | **+** | **+** | **+** |
| Cis-Aconitic acid | - | **+** | **+** | **+** | **+** | - |
| Citric acid | **+** | **+** | **+** | **+** | **+** | - |
| Formic acid | **+** | **+** | **+** | **+** | **+** | **+** |
| D-Galactonic Acid Lactone | **+** | **+** | **+** | **+** | **+** | **+** |
| D-Galacturonic acid | **+** | - | **+** | - | **+** | **+** |
| D-Gluconic acid | **+** | **+** | **+** | **+** | **+** | **+** |
| D-Glucosaminic acid | **+** | - | **+** | **+** | **+** | **+** |
| D-Glucuronic acid | **+** | **+** | **+** | **+** | **+** | **+** |
| α-Hydroxybutyric acid | **+** | **+** | **+** | **+** | **+** | **+** |
| β-Hydroxybutyric acid | **+** | **+** | **+** | **+** | **+** | **+** |
| γ-Hydroxybutyric acid | **+** | **+** | **+** | **+** | **+** | **+** |
| p-Hydroxy Phenylacetic acid | **+** | **+** | **+** | **+** | **+** | **+** |
| Itaconic acid | - | - | **+** | **+** | **+** | **+** |
| α-Keto Butyric acid | **+** | **+** | **+** | **+** | **+** | **+** |
| α-Keto Glutaric acid | **+** | **+** | **+** | **+** | **+** | **+** |
| α-Keto Valeric acid | **+** | **+** | **+** | **+** | **+** | **+** |
| D,L-Lactic acid | **+** | **+** | **+** | **+** | **+** | **+** |
| Malonic acid | - | - | - | **-** | - | - |
| Propionic acid | **+** | **+** | **+** | **+** | **+** | - |
| Quinic acid | **+** | **+** | **+** | **+** | **+** | **+** |
| D-Saccharic acid | **+** | **+** | **+** | **+** | **+** | **+** |
| Sebacic acid | **+** | **+** | **+** | **+** | **+** | **+** |
| Succinic Acid | **+** | **+** | **+** | **+** | **+** | **+** |
| Bromosuccinic acid | **+** | **+** | **+** | **+** | **+** | **+** |
| Succinamic acid | **+** | **+** | **+** | **+** | **+** | **+** |
| Glucuronamide | **+** | **+** | **+** | **+** | **+** | **+** |
| L-Alaninamide | **+** | **+** | **+** | **+** | **+** | **+** |
| D-Alanine | **+** | **+** | **+** | **+** | **+** | **+** |
| L-Alanine | - | - | **+** | **+** | **+** | - |
| L-Alanyl-glycine | **+** | **+** | **+** | **+** | **+** | **+** |
| L-Asparagine | **+** | **+** | **+** | **+** | **+** | **+** |
| L-Aspartic Acid | **+** | **+** | **+** | **+** | **+** | **+** |
| L-Glutamic acid | **+** | **+** | **+** | **+** | **+** | **+** |
| Glycyl-L-Aspartic acid | **+** | **+** | **+** | **+** | **+** | **+** |
| Glycyl-L-Glutamic acid | **+** | - | **+** | **+** | **+** | **+** |
| L-Histidine | - | - | - | - | - | - |
| Hydroxy-L-Proline | - | - | - | - | - | - |
| L-Leucine | **+** | **+** | **+** | **+** | **+** | **+** |
| L-Ornithine | - | - | - | - | - | - |
| L-Phenylalanine | **+** | **+** | **+** | **+** | **+** | **+** |
| L-Proline | **+** | **+** | **+** | **+** | **+** | **+** |
| L-Pyroglutamic acid | **+** | **+** | **+** | **+** | **+** | **+** |
| D-Serine | **+** | **+** | **+** | **+** | **+** | **+** |
| L-Serine | - | - | **+** | **+** | - | **+** |
| L-Threonine | **+** | **+** | **+** | **+** | **+** | **+** |
| D,L-Carnitine | - | - | - | - | - | - |
| γ-Amino Butyric acid | - | - | - | - | - | **-** |
| Urocanic Acid | **+** | **+** | **+** | **+** | **+** | - |
| nosine | - | + | **-** | - | - | **+** |
| Uridine | - | - | - | - | - | - |
| Thymidine | - | - | - | - | - | - |
| Phenyethyl-amine | - | - | - | - | - | - |
| Putrescine | - | - | - | - | - | - |
| 2-Aminoethanol | - | - | - | - | - | - |
| 2,3-Butanediol | - | - | - | - | - | - |
| Glycerol | **+** | **+** | **+** | **+** | **+** | **+** |
| D,L-α-Glycerol Phosphate | - | - | - | **-** | - | - |
| α-D-Glucose-1-Phosphate | - | - | - | - | - | - |
| D-Glucose-6-Phosphate | - | - | - | - | - | - |

+, positive response; -, negative response
